# Modulation of Zn Ion Toxicity in *Pisum sativum* L. by Phycoremediation

**DOI:** 10.3390/plants14020215

**Published:** 2025-01-14

**Authors:** Zornitsa Karcheva, Zhaneta Georgieva, Svetoslav Anev, Detelina Petrova, Momchil Paunov, Miroslava Zhiponova, Ganka Chaneva

**Affiliations:** 1 Department of Plant Physiology, Faculty of Biology, Sofia University, 8 Dragan Tsankov Bul., 1164 Sofia, Bulgaria; zpgeorgiev@uni-sofia.bg (Z.G.); detelina@biofac.uni-sofia.bg (D.P.); 2 Department Dendrology, Faculty of Forestry, University of Forestry, 10 Sveti Kliment Ohridski Blvd., 1756 Sofia, Bulgaria; svetoslav.anev@ltu.bg; 3 Department of Biophysics and Radiobiology, Faculty of Biology, Sofia University, 1164 Sofia, Bulgaria; m_paunov@uni-sofia.bg

**Keywords:** bioaccumulation, heavy metals, microalgae, pea

## Abstract

Microalgae offer a promising alternative for heavy metal removal, and the search for highly efficient strains is ongoing. This study investigated the potential of two microalgae, *Coelastrella* sp. BGV (Chlorophyta) and *Arthronema africanum* Schwabe & Simonsen (Cyanoprokaryota), to bind zinc ions (Zn^2^⁺) and protect higher plants. Hydroponically grown pea (*Pisum sativum* L.) seedlings were subjected to ZnSO_4_ treatment for 7 days in either a nutrient medium (Knop) or a microalgal suspension. The effects of increasing Zn^2^⁺ concentrations were evaluated through solution parameters, microalgal dry weight, pea growth (height, biomass), and physiological parameters, including leaf gas exchange, chlorophyll content, and normalized difference vegetation index (NDVI). Zinc accumulation in microalgal and plant biomass was also analyzed. The results revealed that microalgae increased pH and oxygen levels in the hydroponic medium while enhancing Zn accumulation in pea roots. At low ZnSO_4_ concentrations (2–5 mM), microalgal suspensions stimulated pea growth and photosynthetic performance. However, higher ZnSO_4_ levels (10–15 mM) caused Zn accumulation, leading to nutrient deficiencies and growth suppression in microalgae, which ultimately led to physiological disturbances in peas. *Coelastrella* sp. BGV exhibited greater tolerance to Zn stress and provided a stronger protective effect when co-cultivated with peas, highlighting its potential for phycoremediation applications.

## 1. Introduction

Heavy metals and metalloids play a key role in triggering stress response reactions in land plants and algal biosystems [1]. Excessive heavy metals are among the most significant pollutants, negatively impacting crop growth and yield, and pose a critical challenge to food security and environmental sustainability [2,3]. Effective and sustainable wastewater treatment can be achieved not only by removing macronutrients and heavy metals from the polluted environment [4,5] but also by remediation of toxic contaminants such as pesticides, which, even in minimal amounts, can severely affect human health and freshwater ecosystems [6]. One approach to mitigating environmental pollution is bioremediation, an economical and effective method for removing heavy metals from various environments, particularly wastewater [7]. In bioremediation, living or dead organisms (e.g., bacteria, fungi, algae) are used to reduce and remove environmental pollution through metabolic processes [8]. The use of microalgae for phycoremediation is one of the most promising modern strategies and is considered an innovative alternative to conventional techniques. Phycomediation offers several advantages over other traditional physicochemical approaches, such as ion exchange, dialysis and electrodialysis, adsorption with activated carbon, chemical reduction or oxidation, etc. In particular, the microalgae provide more efficient pollutant removal, lower technical implementation costs, and reduced maintenance requirements [9]. Traditional processing methods often require significant resources and generate considerable toxic by-products. In contrast, phycoremediation is an eco-friendly approach that utilizes a variety of non-pathogenic algae, including *Spirulina* sp., *Chlorella* sp., *Chlamydomonas* sp., *Oscillatoria* sp., *Nostoc* sp., and *Scenedesmus* sp. [10].

Microalgae can assimilate considerable amounts of nutrients (carbon, nitrogen, and phosphorus) from wastewater to meet their growth and development needs [11]. Furthermore, they can thrive under various climatic and environmental conditions [12], making them a promising alternative for wastewater treatment [13]. Another benefit of microalgae is their ability to increase dissolved oxygen (O_2_) availability in the environment, aiding in the extraction of nutrients and toxic pollutants [14,15]. During photosynthesis, microalgae can remove excess carbon dioxide from the environment, leading to an increase in pH. This pH adjustment is beneficial in bioremediation processes, as some pollutants are more soluble and easily removed under more alkaline pH conditions.

Zinc (Zn), a metal found in soil and water bodies, enters through both natural and anthropogenic activities. Sewage sludge is the main source of pollution of agricultural land with Zn [16,17]. Zn is released as bivalent cations (Zn^2^⁺) and forms soluble salts, including sulphates, nitrates, acetates, etc.; less soluble Zn-ammonium phosphate, Zn hydroxide and Zn carbonate; and a range of soluble and insoluble organic complexes [18]. Excessive levels of Zn^2^⁺ can disrupt soil and water microbial diversity, affect the bioavailability and absorption of other essential metals, and cause acute toxicity [19]. Elevated Zn^2^⁺ concentrations are phytotoxic, leading to species-specific structural and functional abnormalities in plants that weaken their performance [20,21]. However, Zn is also a plant nutrient that is essential for photosynthesis and carbohydrate metabolism [22]. It is also a critical component in the carbonic anhydrase process, which catalyzes the dehydration of carbonic acid and facilitates the incorporation of carbon dioxide. Recent studies demonstrate that treating Zn^2+^-containing wastewater with microalgae provides dual benefits: pollutant reduction/removal and simultaneous production of biodiesel from metal-resistant biomass [23]. The uptake (biosorption) of Zn^2^⁺ by microalgae occurs by binding to the functional groups of lipids, carbohydrates, and proteins found in the microalgal cell walls [24]. The co-cultivation of plants and microalgae represents an optimized bio-system that enhances plant performance while simultaneously reducing environmental nutrient loads, ultimately contributing to sustainable agriculture. Hydroponically grown plants can have higher yields and better quality [25]. The green pea (*Pisum sativum* L., Fabaceae) is a legume with high nutritional value for humans and animals, and it is considered an affordable protein source (on average, 20-22% protein per seed), essential amino acids (lysine and leucine), and vitamins and minerals like phosphorus (P), potassium (K^+^), calcium (Ca^2+^), copper (Cu^2+^), iron (Fe^2+^), and Zn^2+^ ions [26,27,28]. Its adaptability allows it to grow in various environments, including soils and hydroponic systems under heavy metal stress. Therefore, understanding the effects of Zn toxicity on essential food crops like peas is of urgent importance.

Previous reports were focused on microalgal dual function: phycoremediation of Zn and other heavy metals, and production of feed additives or biodiesel [17,23]. Karcheva et al. [29] demonstrated that the fast-growing Bulgarian green alga *Coelastrella* sp. BGV and the desert-dwelling cyanoprokaryote *Arthronema africanum* Schwabe & Simonsen have high absorption capacity for lead ions (Pb^2+^), and, to a lesser extent, for Cu^2+^ and cadmium (Cd^2+^) ions. The present study aimed to elucidate the physiological mechanisms of the response of these two little-studied strains of microalgae and pea plant co-cultivation systems exposed to elevated Zn^2+^ concentrations. To understand adaptations to Zn stress, we monitored pH and O_2_ levels in the hydroponic systems, the growth rates of microalgae and pea plants, and the photosynthetic parameters of the plants. Zn accumulation was evaluated in the microalgae and in the aerial parts and roots of the pea plants.

## 2. Results

### 2.1. Microalgae/Pea Plant Co-Cultivation Under Zn Treatment

**Figure 1:**
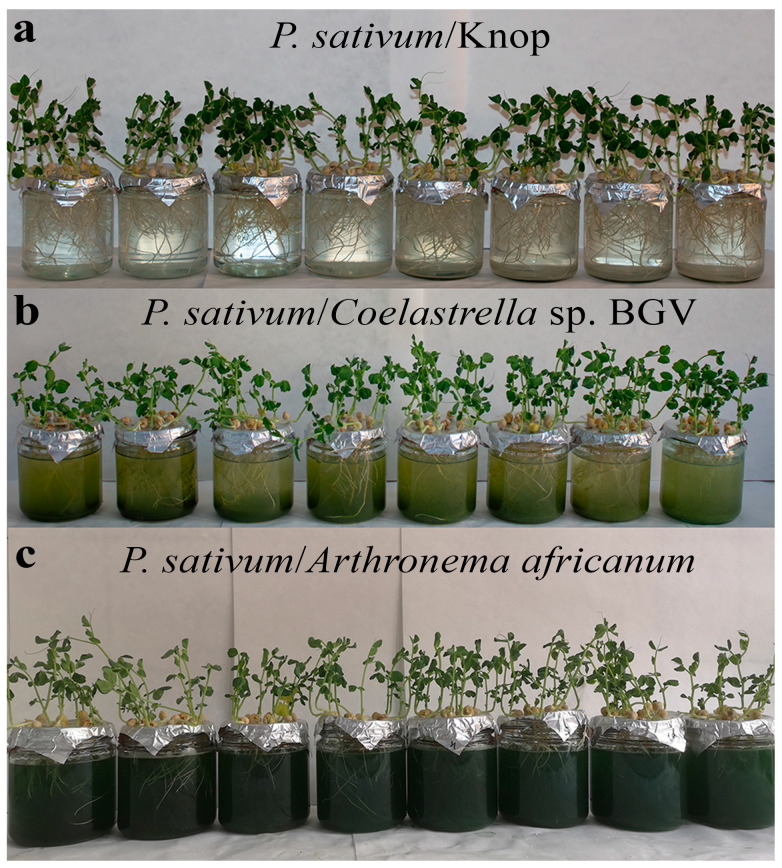
Experimental co-cultivation systems under ZnSO_4_ treatment on day 3. (**a**) *P. sativum* in control Knop medium. (**b**) *P. sativum* with *Coelastrella* sp. BGV. (**c**) *P. sativum* with *Arthronema africanum*.

The effect of Zn was monitored in three experimental systems in which pea plants were cultivated using (i) control Knop’s solution; (ii) *Coelastrella* sp. BGV; and (iii) *Arthronema africanum*. The algal suspensions were maintained in the exponential growth phase (Figure 1).

The experiment lasted 7 days, with analyses performed on days 0, 3, 5, and 7. A series of ZnSO_4_ concentrations (2 to 15 mM) were tested for comparison with untreated control.

#### 2.1.1. pH Levels in Nutrient Solutions

The pH levels varied significantly among the three experimental systems. In the control Knop’s solution system, pH values remained constant throughout the experiment, maintaining a level of approximately 6. In the system treated with the green algae *Coelastrella* sp. BGV, the pH increased to 9 on days 3, 5, and 7. However, a decrease in pH was observed at ZnSO_4_ concentrations of 10 and 15 mM by the end of the experiment, although these values were still higher than in the control. In the presence of *Arthronema africanum*, the pH increased by 22% in the untreated control variant. A slight increase was also reported at 2 mM ZnSO_4_. In the remaining variants, the pH decreased over the course of the experiment reaching the control levels (Table 1).

#### 2.1.2. O_2_ Content in Nutrient Solutions

The average O_2_ content in the three experimental systems was approximately 6.5 mg/L at the start of the experiment, and increased up to 7.5–8.7 in the presence of microalgae (Table 2). High Zn concentrations (10–15 mM) caused a general reduction in O_2_ levels, reaching 5 to 6 mg/L at day 7. In contrast, at lower Zn concentrations (2 and 5 mM), O_2_ levels increased in the microalgae systems during co-cultivation, reaching 7 to 8 mg/L. At these lower Zn concentrations, *Arthronema africanum* produced more O_2_ than *Coelastrella* sp. BGV.

### 2.2. Effect of Zn on Growth Parameters of Microalgae and Higher Plant

#### 2.2.1. Effect of Zn Stress on Algal Biomass

**Figure 2:**
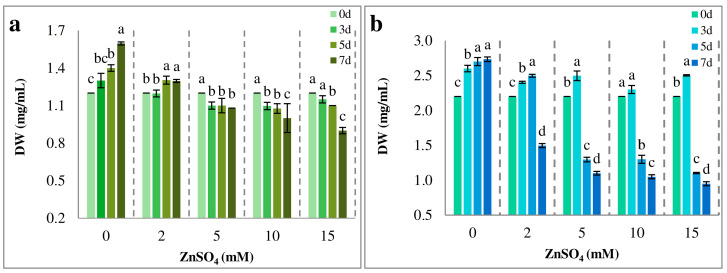
Effect of Zn treatment on microalgal growth. Samples were analyzed at 0, 3, 5, and 7 days (d). The data are represented as mean ± SE. Different letters indicate statistically significant differences in dry weight (DW) for each treatment at *p* < 0.05. (**a**) *Coelastrella* sp. BGV. (**b**) *Arthronema africanum*.

The growth of algal strains following treatment with Zn, supplied to the culture solution as ZnSO_4_, was determined by measuring the changes in biomass accumulation. Growth stimulation of *Coelastrella* sp. BGV was observed at 0 and 2 mM ZnSO_4_, particularly pronounced on days 5 and 7 from the start of treatment. The presence of ZnSO_4_ caused an inhibitory effect on the growth of *Coelastrella* sp. BGV at 5 mM (Figure 2a). At higher ZnSO_4_ concentrations, the inhibitory effect was more evident. Notably, at 15 mM ZnSO_4_, growth inhibition of approximately 25% was recorded on day 7 compared to the control.

The growth of *Arthronema africanum* was severely reduced by 2 mM ZnSO_4_ on day 7 (Figure 2b). Growth inhibition became notable at higher concentrations after 5 and 7 days of cultivation. At 5 and 10 mM ZnSO_4_, biomass decreased by 52% and 54%, respectively, compared to the control on day 0. At 15 mM ZnSO_4_ on day 7, the maximum biomass reduction of 58% was recorded.

The experimental results demonstrated that treatment of both algal strains with the selected ZnSO_4_ concentrations did not severely affect growth on day 3, suggesting a level of resistance to the Zn impact. However, a significant decrease in growth was observed in both strains at ZnSO_4_ concentrations of 5, 10, and 15 mM on days 5 and 7. Interestingly, the green alga *Coelastrella* sp. BGV exhibited greater tolerance compared to *Arthronema africanum*.

#### 2.2.2. Effect of Zn Stress on Pea Growth

**Figure 3:**
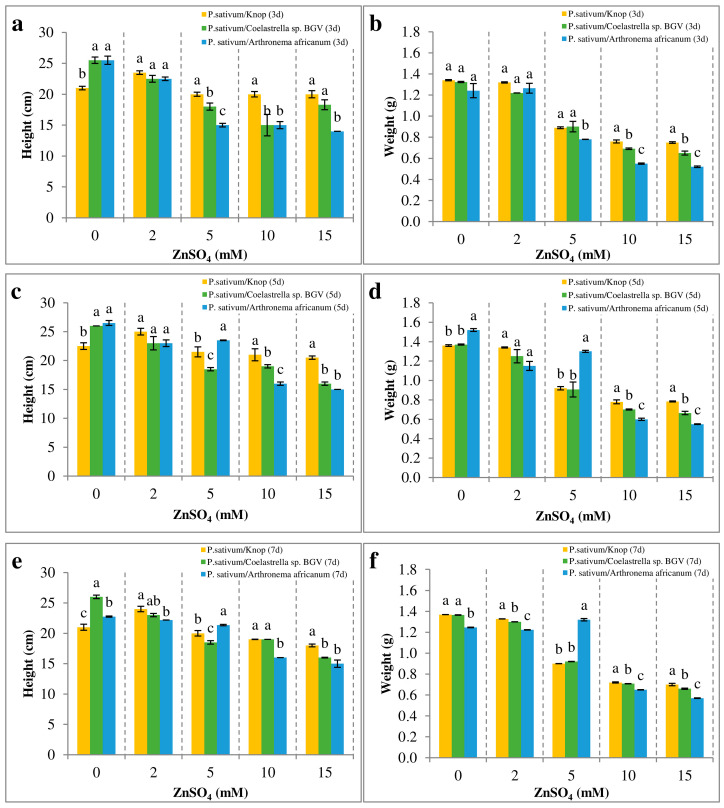
Effect of Zn treatment on *P. sativum* morphometric indicators. The three experimental systems with pea plants were subjected to a series of concentrations of ZnSO_4_ for seven days. The data are represented as mean ± SE. Different letters indicate statistically significant differences between experimental systems for each treatment at *p* < 0.05. (**a**,**c**,**e**) Plant height at days 3, 5, 7. (**b**,**d**,**f**) Biomass (fresh weight) at days 3, 5, 7.

In the control Knop medium, no significant changes in the height of pea plants were observed at any of the tested concentrations throughout the entire cultivation period (Figure 3a,c,e).

Notably, the presence of microalgae had a favorable influence on *P. sativum* growth, with a nearly 20% elongation-promoting effect. The application of ZnSO_4_ concentrations exceeding 2 mM in the medium caused a general reduction in plant height as early as day 3, and the presence of either microalgae strain did not have an ameliorating effect.

The fresh biomass of pea plants generally correlated with the plant height (Figure 3b,d,f). An interesting observation was made on days 5 and 7 in the *Arthronema africanum* system with 5 mM ZnSO_4_, where a 30% growth stimulation of pea plants was recorded compared to the other two experimental systems.

Overall, the analysis of growth parameters indicated that the co-cultivation of *Coelastrella* sp. BGV and *P. sativum* had a more prominent positive effect in mitigating the adverse impact of high Zn concentrations compared to the co-cultivation of *P. sativum* with the cyanoprokaryotic strain.

### 2.3. Investigation of Key Physiological Parameters in Pea Plants Following Zn Treatment

**Figure 4:**
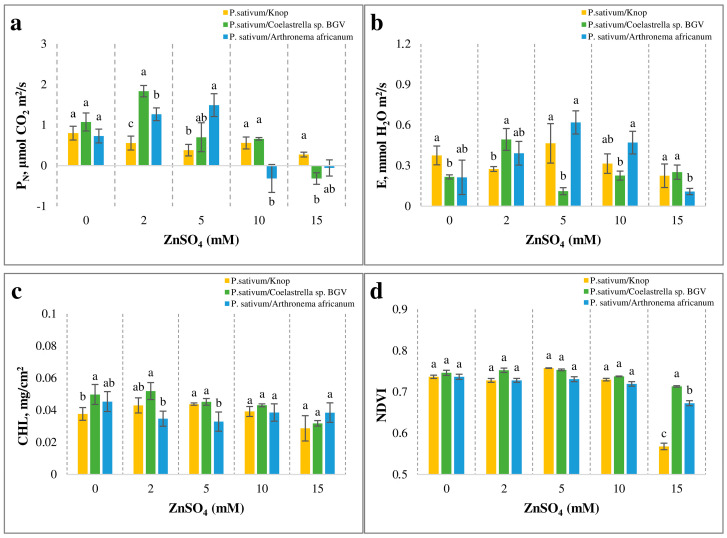
Effect of Zn treatment on physiological indicators in *P. sativum* leaves. The three experimental systems with pea plants were subjected to a series of concentrations of ZnSO_4_ for five days. The data are represented as mean ± SE. Different letters indicate statistically significant differences between experimental systems for each treatment at *p* < 0.05. (**a**) Net photosynthetic rate (P_N_). (**b**) Transpiration rate (E). (**c**) Chlorophyll (CHL) content. (**d**) Normalized difference vegetation index (NDVI).

To evaluate the physiological changes in *P. sativum* upon treatment with ZnSO_4_, photosynthesis-related parameters were analyzed on day 5 (Figure 4).

#### 2.3.1. Leaf Gas Exchange Parameters

In the control experimental system (*P. sativum*/Knop), there were no significant differences in the levels of carbon balance in pea leaves (Figure 4a). The highest net photosynthetic rate (P_N_) was recorded at 2 mM ZnSO_4_ during co-cultivation with the microalgae. *Coelastrella* sp. BGV provided a protective effect, resulting in an approximately 2-fold increase of P_N_, while *Arthronema africanum* induced a 1.5-fold increase. At 5 mM ZnSO_4_, the positive effect of the microalgae persisted, with *Arthronema africanum* significantly enhancing pea photosynthesis, which was in accordance with the increase in plant biomass (Figure 3d). At the highest ZnSO_4_ concentrations (10 and 15 mM), the microalgae had no significant effect on, or even inhibited, pea photosynthesis relative to the control. In summary, a stimulating effect of low Zn concentration was only observed with added microalgae but not in Knop. At high Zn concentration, however, a negative carbon balance was observed in the presence of microalgae, but again, not in Knop.

The transpiration rate (E) of *P. sativum* was not severely affected by ZnSO_4_ in the *P. sativum*/Knop control system (Figure 4b). A positive effect was observed during co-cultivation with microalgae at 2 mM ZnSO_4_, resulting in a 30–40% increase of E. In *Arthronema africanum*, a similar stimulation was observed in a wider range of concentrations (5 and 10 mM ZnSO_4_). At 15 mM ZnSO_4_, the effect of *Coelastrella* sp. BGV stabilized near control levels, whereas *Arthronema africanum* caused a reduction in E.

The data suggests that the two elements of gas exchange (photosynthesis and transpiration) could be influenced by stomatal conductance in pea leaves, which seem to be affected by the concentration of Zn^2+^ and the microalgal suspensions, while it was absent in Knop. As shown in Table 1, the pH levels in *P. sativum*/Knop are rather constant during all treatments, while the pH during co-cultivation of pea in microalgal suspensions is more dynamic and could influence the assimilation of mineral nutrients and respectively affect plant physiological responses.

#### 2.3.2. Total Chlorophyll Content

The total chlorophyll content in pea leaves across all variants of the three experimental systems was not significantly affected after treatment with up to 10 mM ZnSO_4_ (Figure 4c). Interestingly, at the lower Zn^2+^ doses, a slight increase in chlorophyll content was observed in variants of *P. sativum* co-cultivated with *Coelastrella* sp. BGV, compared to those co-cultivated with *Arthronema africanum*. At 15 mM ZnSO_4_, *P. sativum* in the experimental system with *Coelastrella* sp. BGV showed a decrease compared to the other treatments. The most notable drop in chlorophyll levels was recorded in plants treated with 2 and 5 mM ZnSO_4_ in the *P. sativum/Arthronema africanum* system, showing a 25% reduction compared to control plants grown in Knop’s solution with *P. sativum*. As shown in Figure 3d,f, at 5 mM ZnSO_4_, *Arthronema africanum* boosted pea growth, which could include new leaf formation and chlorophyll synthesis.

#### 2.3.3. Normalized Difference Vegetation Index

The Normalized Difference Vegetation Index (NDVI), an indicator of the plant physiological state, displayed similar values across the tested systems (Figure 4d). An exception was observed at 15 mM ZnSO_4_, when a sharp 78% drop in NDVI was measured in the control *P. sativum*/Krop system compared to the other ZnSO_4_ concentrations. In contrast, NDVI values remained relatively high in the presence of *Coelastrella* sp. BGV and *Arthronema africanum*, indicating successful adaptation of pea plants in microalgal co-cultivation systems under Zn-induced stress.

### 2.4. Accumulation of Zn in the Biomass of Microalgae and Higher Plants

#### 2.4.1. Accumulation of Zn in the Biomass of Experimental Algal Species

The physiological changes observed in *Coelastrella* sp. BGV and *Arthronema africanum* suggest that Zn influences metabolic processes in algal cells through distinct mechanisms. This is supported by the differences in their ability to accumulate heavy metals from the nutrient medium. Both microalgae exhibited a high capacity for metal ion absorption, with *Arthronema africanum* showing a greater tendency to accumulate Zn in its biomass compared to *Coelastrella* sp. BGV (Table 3 and Table 4).

The accumulation of Zn showed a direct negative effect on the DW of *Coelastrella* sp. BGV (*r* = −0.988; *p* = 0.01). On the other hand, the algal biomass demonstrated strong positive correlation with elements such as Fe (*r* = 0.985; *p* = 0.002), Cu (*r* = 0.980; *p* = 0.003), Mg (*r* = 0.958; *p* = 0.01), and Ca (*r* = 0.973; *p* = 0.005). The latter elements seem to contribute to the pea fresh weight, as well (*r* ≥ 0.9, *p* > 0.05). Interestingly, in *Coelastrella* sp. BGV, the accumulation of Zn showed the strongest reverse correlation with Mn (*r* = −0.985; *p* = 0.02), and Mn seemed essential for pea fresh weight (*r* = 0.913; *p* = 0.03) and P_N_ (*r* = 0.901; *p* = 0.04).

In accordance with the inhibition of the growth of *Arthronema africanum* (Figure 2b), a significant correlation was established with the accumulation of Zn (*r* = −0.998; *p* = 0.0001) and P (*r* = −0.996; *p* = 0.0003). In parallel, the reduction in algal biomass was interlinked with decreasing levels of Mn (*r* = 0.978; *p* = 0.004), Mg (*r* = 0.958; *p* = 0.01), and Ca (*r* = 0.990; *p* = 0.001). A sharp drop in the content of these elements was observed at 5 and 10 mM ZnSO_4_ (Table 4), which is the breakpoint of boosting pea growth, as shown by the increase in the pea fresh weight (Figure 3d,f) and P_N_ (Figure 4a). Curiously, the microalga contained significantly more Cu and Mn than *Coelastrella* sp. BGV.

#### 2.4.2. Accumulation of Zn in the Biomass of *P*. *sativum*

In the control variants, both algae competed for Zn with pea roots, whose reduced accumulation correlated with the positive effect on plant growth observed in Figure 3. Excessive concentrations of ZnSO_4_ (10 and 15 mM) caused visible damage to leaves and roots, indicating Zn accumulation. Analysis of Zn distribution in the plant organs revealed a higher accumulation of the metal in the roots compared to the aerial parts across all three experimental systems (Table 5). Overall, the microalgae lowered Zn content in pea aerial parts but elevated it in the roots.

### 2.5. Principal Component Analysis

**Figure 5:**
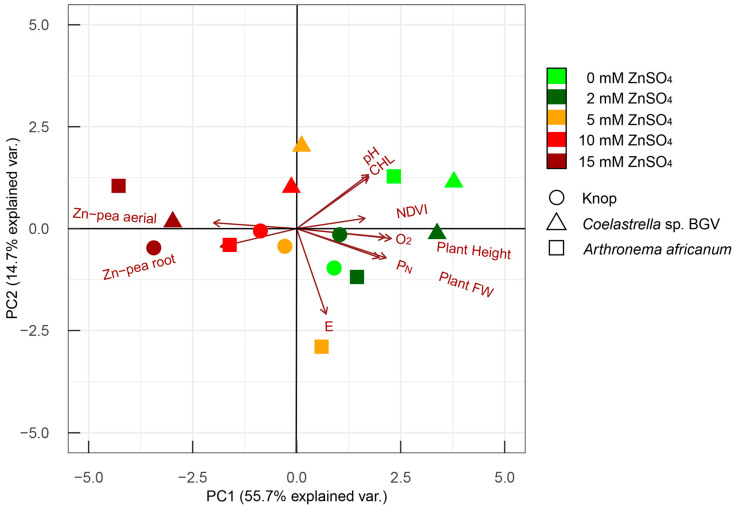
PCA of 10 experimental parameters representing differences between co-cultivation systems under increasing ZnSO_4_. Variants are given as circles, triangles, and squares, colored depending on the applied Zn^2+^ concentration, while parameters are visualized as vectors.

Principal component analysis (PCA) facilitates the visualization of the overall comparison between the experimental variants. Of the total data variance, 70.4% was explained by the first and second principal components (PCs) combined, and 55.7% and 14.7%, respectively (Figure 5).

The length and orientation of a parameter vector in the PC1/PC2 coordinate system reflect the contribution level of the respective parameter to the components: the longer the vector and the higher its proximity to an axis, the greater its contribution to the respective PC. All the parameters but E, pH, and CHL primarily determine PC1, while E – PC2, and pH and CHL, influence both equally. Differences between the individual experimental systems with increasing ZnSO_4_ concentrations are strong and reflected along PC1—its negative values are associated with higher Zn content. The PCA clearly demonstrates that high Zn levels lead to *P. sativum* growth retardation and accumulation of the metal in the root and above-ground part. The Zn protective effect of microalgae co-cultivation was observed for all ZnSO_4_ treatments for *Coelastrella* sp. BGV and up to 5 mM for *Arthronema africanum*.

The PCA revealed a strong positive correlation between O_2_ content in the medium and plant performance (growth—height, fresh weight; and photosynthesis—P_N_, NDVI). The pH and the CHL also showed high correlation, while E appeared uncorrelated to the other parameters.

## 3. Discussion

The increasing ecological danger, coupled with the fine balance between Zn’s essentiality and its toxicity, have drawn significant attention to the metabolic effects of this metal and its role in sustainable agriculture [30,31,32]. Investigating the physiological and biochemical impairments caused by high Zn levels, along with the mechanisms of uptake and transport, is crucial for developing strategies to mitigate Zn toxicity and improve agricultural productivity.

### 3.1. Zn Accumulation in the Algal Biomass

The involvement of microalgae in co-cultivation systems enables the examination of heavy metal impacts and changes in defense mechanisms involved in detoxification processes. Co-cultivation of *P. sativum* with the microalgae *Coelastrella* sp. BGV and *Arthronema africanum* under Zn^2+^ stress conditions, revealed partial stress alleviation. The established protective properties of *Coelastrella* sp. BGV and *Arthronema africanum* likely result from their ability to sequester Zn salts in their biomass. Zn is an essential trace element for microalgae metabolism [33]. Our results demonstrated that the green microalga *Coelastrella* sp. BGV exhibited greater tolerance to elevated Zn^2+^ levels than the cyanoprokaryotic strain over the experimental period. These findings align with previous studies showing that lower doses of micronutrients often promote microalgal growth, while higher doses inhibit it, with effects varying by metal ion and algal strain/species [34]. For instance, Mathimani et AL. [23] reported no visible Zn-induced stress on the green algae *Selenastrum* sp., which showed consistent growth across all Zn^2+^ concentrations tested. Similarly, the phytoplankton *Tetraselmis* sp. Demonstrated high Zn resistance compared to other species [35]. Other studies, such as Santos et al. [36], highlighted *Chlorella vulgaris* Beijerinck’s ability to grow in Zn^2+^-rich environments, supporting its potential for bioremediation. Among cyanoprokaryotes, *Lyngbya taylorii* Drouet & Strickland has been shown to possess a high adsorption capacity for heavy metals (Pb, Cd, nickel/Ni, Zn), simple cultivation, easy separation, and high biomass yield [37]. Parallel analysis of *Arthronema africanum* indicated moderate capability for heavy metal assimilation.

The pH of the culture medium significantly influences metal ion solubility and interactions with biomass functional groups, making it a crucial factor in biosorption [38]. Higher pH levels in the *Coelastrella* sp. BGV system compared to controls likely enhanced Zn^2+^ sorption capacity by reducing competition between protons and metal ions for binding sites [39]. Our results confirmed that both algal strains accumulated significant amounts of Zn from the culture medium, with *Arthronema africanum* exhibiting higher absorption capacity despite lower pH levels. The pH dependence of metal adsorption is largely related to the type and ionic state of the functional groups in the biosorbent. Positively charged hydrogen ions can compete with metal ions for binding to cell wall ligands [40]. An increase in pH results in a lower concentration of protons, reducing the competition between protons and heavy metal ions. Consequently, an increase in the sorption capacity for Zn^2^⁺ ions can be observed [39]. The optimum pH for *L. taylorii* with high heavy metal uptake capacity was between pH 3 and 7 for Pb, Cd, and Zn [37]. In the present work, the two microalgal strains accumulated significant amounts of heavy metal introduced into the culture medium. The increased Zn sorption in *Arthronema africanum* correlated with accumulation of P, which might be due to reduced metabolic activity, or a compensation mechanism associated with growth retardation. In *Coelastrella* sp. BGV, the accumulation of Zn caused a substantial nutrient element deficiency. However, the heavy metal uptake occurred more slowly and the microalgal growth was retained longer, highlighting this alga as a good candidate for phycoremediation. Respectively, *Coelastrella* sp. BGV response to other heavy metal ions (Pb^2^⁺, Cd^2^⁺, and Cu^2^⁺) demonstrated a defense mechanism by increasing the carotenoid content and activity of antioxidant enzymes, such as superoxide dismutase and catalase [29]. The microalgal accumulation of lipids and their release were also shown to occur in parallel with oxidative stress response and play a role in reducing heavy metal toxicity [17,20,24,41].

### 3.2. Impact of Zn Toxicity on Plant Performance

In our experimental setup, in the co-cultivation systems at lower Zn^2+^ concentrations, the positive effect on *P. sativum* growth correlated with the increased O_2_ content in the medium. Under the higher 10 and 15 mM ZnSO_4_ treatments, reduced and even negative net photosynthesis rates in *P. sativum* were observed. Interestingly, transpiration rate values at low Zn^2+^ levels corroborated with ameliorated plant performance; however, upon high doses of the heavy metal, an impairment was observed with respect to the low photosynthetic rates. Excess Zn disturbs water uptake and stomatal function, leading to reduced photosynthesis and biomass [32]. Zn toxicity is known to minimize net photosynthesis, although the underlying mechanisms—whether related to stomatal or mesophyll conductance, photochemistry, or biochemistry—remain unclear [39,42,43]. For instance, Misra et al. [42] observed in *Pelargonium graveolens* L’Hér. that Zn treatment restricted stomatal conductance, limiting CO₂ diffusion into leaves and inhibiting photosynthesis. Fatemi et al. [43] also linked high Zn^2+^ concentrations in *Brassica rapa* L. to reduced stomatal conductance, root reduction, and decreased photosynthetic rates. We speculate that over time, mineral deficiency in the plants could occur, as observed in the algal biomass by the gradual decline in essential elements (Fe, Cu, Mn, Mg, Ca).

### 3.3. Plant Tolerance to Zn Stress

Andrejić et al. [44] demonstrated that *Miscanthus* × *giganteus* Greef et Deu. accumulates Zn differently across plant organs. Despite Zn-induced reductions in gas exchange, stomatal conductance, and photosynthesis, the plant maintained high photosynthetic capacity under Zn stress, reflecting its tolerance. Similarly, *P. sativum* displayed resilience under excessive Zn^2+^ concentrations, with co-cultivation systems partially mitigating Zn toxicity. The constant CHL content and NDVI values across all Zn^2+^ concentrations suggest delayed toxicity in the presence of microalgae. *Coelastrella* sp. BGV and *Arthronema africanum* provided a more alkaline and O_2_-enriched medium for plant growth. In addition, microalgae are expected to release metabolites that contribute to plant growth under unfavorable conditions [17,20,24,41]. Interestingly, *P. sativum* alone also demonstrated heavy metal adaptation. First, this legume plant is known for its antioxidant activity due to the existence of various bioactive components (polyphenols, polysaccharides, and peptides) [45]. Second, it is likely that the plant releases molecules (flavonoids and root exudates) to attract the microalgae for phytoprotection [46]. Third, the predominant Zn accumulation in pea roots suggests an additional tolerance mechanism under heavy metal stress conditions by limiting the transportation towards the photosynthetic organs.

### 3.4. Future Perspectives

Microalgae comprise an incredibly diverse group, encompassing thousands of genera and species, each with unique characteristics and applications. Exploring and utilizing this diversity is essential for unlocking their potential across various industries and environmental initiatives [15]. Our future investigations would be directed towards further elucidation of the mechanisms of Zn bioaccumulation in the studied algae strains and their phytoprotective role. Accordingly, we plan to clarify the mechanism of the algal response to Zn^2+^ by estimation of oxidative stress, enzymatic antioxidant activity, and accumulation of pigments and metabolites and their release in the environment. Integration of advanced technologies, such as omics approaches (genomics, transcriptomics, proteomics, and metabolomics), will provide a deeper understanding of microalgae’s molecular responses to pollutants. This knowledge will enable the identification of key signaling pathways involved in bioremediation processes.

More studies report on the development of monitoring approaches based on bacterial or algal test systems that could serve as bioassays for testing toxic chemicals (pure chemicals and chemical mixtures) in an aquatic environment [47,48]. The studied microalgae, especially the procaryotic *Arthronema africanum*, prove to have the potential for screening the effects of other heavy metals, herbicides, etc., and developing standard protocols for risk assessment and optimization of bioremediation strategies.

## 4. Materials and Methods

### 4.1. Cultivation Conditions

#### 4.1.1. Microalgal Material and Cultivation

The green alga *Coelastrella* sp. BGV, a newly isolated Bulgarian strain [49], was grown at 28 °C, on the Šetlik nutrition medium [50] modified after Georgiev et al. [51]. The cyanoprokaryote *Arthronema africanum* Schwabe & Simonsen, strain Lukavsky, 1981/01 (Komárek and Lukavský 1988) CCALA (Tøeboò Collection of Autotrophic Organisms, Czech Republic) was cultivated at 28–30 °C, on the nutrition medium based on the Allen and Arnon [52] and Zehnder [53] media, modified by Chaneva et al. [54]. Both strains were intensively cultivated in 200 mL vessels, under continuous illumination by white light, 150 μmol photons/m^2^/s. The carbon source was provided by bubbling sterile 2% (*v*/*v*) CO_2_ in air (100 L/h). The algal cultures were centrifuged at the end of the exponential growth phase, and the biomass was further resuspended in a fresh nutrition medium at an inoculum 0.5 mg/mL DW (dry weight).

#### 4.1.2. Plant Material and Cultivation

Pea plants (*P. sativum* L.), variety RAN*1*, were used as a model object. The seeds were incubated in the dark at 25 °C for germination. The pea sprouts were placed in glass vessels with a volume of 350 mL with Knop nutrient medium (Knop nutritive solution has the following chemical composition in g/L: Ca(NO_3_)_2_ 0.25 g, KH_2_PO_4_ 0.25 g, MgSO_4_ 0.25 g, KCl 0.25 g, and traces of FeSO_4_), at a photoperiod of 16/8 h, a temperature of 25 °C, and an illumination of 150 µmol photons/m^2^/s. Ten-day-old pea plants (at the 3-leaf developmental stage) were treated in the same glass vessels.

#### 4.1.3. Experimental Systems

The treatment of *P. sativum* was performed when the plants were at the 3-leaf developmental stage. At this stage, microalgae and ZnSO_4_ were added for seven days. Three experimental systems were set up where *P. sativum* was *i*. grown in Knop medium; *ii*. co-cultivated with the green alga *Coelastrella* sp. BGV; *iii*. co-cultivated with the Cyanoprokaryote *Arthronema africanum*. The microalgal suspensions were in their stationary phase of development. The effect of Zn on the three experimental systems was compared by applying a series of concentrations of ZnSO_4_ (0 to 15 mM). Samples for analyses were taken on days 3, 5, and 7 during treatment.

#### 4.1.4. Experimental Medium Characteristics

The pH was evaluated by a sensor combination electrode (Mettler Toledo LE 438, Zaventem, Belgium). The O_2_ concentration in the solutions was quantified by an O_2_ electrode (XS Oxy 70 Vio portable oximeter, Carpi, MO, Italy). The measurements of these two parameters were performed in dynamics (0, 3, 5, 7 days) for the three experimental systems.

### 4.2. Morphological Parameters

#### 4.2.1. Growth of Microalgal Strains

The growth of the algal culture was tracked by biomass, according to the increase in dry weight (DW). For this purpose, 5 mL of the algal suspension was placed in tared centrifuge tubes, and 0.5 mL of 11.5% CH_3_COOH was added. After 20 min of incubation, the tubes were centrifuged at 8000 rpm for 10 min and the precipitate was dried at 105 °C to constant weight.

#### 4.2.2. Growth of Pea Plants

Morphometric indicators of peas’ growth dynamics (3, 5, 7 days) under treatment with ZnSO_4_ in the three experimental systems were monitored. The height and fresh weight of pea plants were recorded. The experiment was carried out in three biological repetitions (*n* ≥ 6).

### 4.3. Leaf Gas Exchange

The leaf gas exchange parameters—net rate of photosynthesis (P_N_, µmol CO_2_ m^−2^ s^−1^), and transpiration rate (E, mmol H_2_O m^−2^ s^−1^)—were recorded by an infrared gas analyzer system Li6400 (LI-COR Biosciences Inc., Lincoln, NE, USA) equipped with a light-source chamber (LI6400-02). Here, 10 L buffer was used to counterpoise the fluctuations of CO_2_ and H_2_O in the air [55]. The second fully expanded leaf was used to read leaf gas exchange parameters. The leaves were preliminary adapted to the surrounding environment. Records were completed between 09:00 h and 12:00 h under controlled conditions: temperature, 25 °C; relative air humidity, 45 to 55%; air flow rate, 250 µmol/s; actinic PAR 1000 μmol m^2^/s photon flux density.

### 4.4. Leaf Pigment Content

Total chlorophyll content in fully developed leaves was assessed by a non-destructive method using a portable chlorophyll meter atLEAF^+^ (FT Green LLC, Wilmington, DE, USA) that estimates the intensity of the green color of the leaves at two wavelengths: 653 nm (red color) and 931 nm (infrared color) [56].

### 4.5. NDVI

The normalized difference vegetation index (NDVI) was determined with a portable meter PlantPen NDVI-300 (PSI Corporation, Drásov, Czech Republic). The Plant-Pen NDVI-300 m measures the leaf absorbance or reflectance difference between red and near infra-red light, and this measure strongly correlated to the total chlorophyll of leaf [56,57,58].

### 4.6. Accumulation of Mineral Elements

To measure Zn in plant and algal biomass, and the elements Fe, Cu, Mn, Mg, Ca, and P in the algae, a dry burn at 450 °C was performed, followed by dissolving in mineral acid (20% HCl). The above elements, except P, were analyzed using an Atomic absorption spectrophotometer (Perkin Elmer M-5000, Wellesley, MA, USA) and using standards. The measurement of P was done colorimetrically and via standards.

### 4.7. Statistical Analysis

All measurements included plant and algal material from three biological replicates, including three technical replicates (*n* ≥ 3). Each biological replicate contained plant material from an average of 10 plants (*n* ≥ 10), and the biological replicate for algae was from an average of 3 samples (*n* ≥ 3). The results are presented as means ± standard errors (SE). One-way ANOVA followed by Fisher’s multiple comparison test was used to statistically evaluate experimental variants. Correlation analysis was performed by SigmaPlot 11.0. Principal component analysis (PCA) of 10 parameters values for all the examined variants was performed by the *prcomp* function from the *stats* package in R 4.4.1 programming language, with centering to zero and scaling to unit variance of the experimental values. PCA graphs were plotted by the R package *ggbiplot* 0.55.

## 5. Conclusions

Our study confirms that using microalgae for phycoremediation is a reliable approach to mitigating heavy metal stress. The two types of microalgae tested exhibit species-specific stress responses. The cyanoprokaryote *Arthronema africanum* is much more sensitive to Zn stress but accumulates the heavy metal to a greater extent compared to *Coelastrella* sp. BGV, which demonstrates higher tolerance to the toxic effects of Zn. We suggest that further studies could prove *Coelastrella* sp. BGV as a suitable species for the non-toxic and effective removal of heavy metals.

In the co-cultivation system involving *P. sativum* and *Coelastrella* sp. BGV, a certain modulation of Zn toxicity was observed, reflected in the beneficial impact of the green microalga on the physiological activity of pea plants. We propose that the water-soluble suspension and the release of metabolites into the cultivation medium by *Coelastrella* sp. BGV created more favorable conditions for the growth and development of pea plants in a Zn-contaminated environment. Additionally, the growth analysis of *P. sativum* suggested that the plant itself adapts to some extent to stress probably by exerting its antioxidative activity and by retaining Zn transport into the roots.

## Figures and Tables

**Table 1:** Levels of pH in *P. sativum* experimental systems exposed to a series of Zn concentrations. Samples were analyzed at 0, 3, 5, and 7 days of co-cultivation.

pH	*P. sativum*/ Knop				*P. sativum*/ *Coelastrella* sp. BGV				*P. sativum*/ *Arthronema africanum*			
ZnSO_4_	0d	3d	5d	7d	0d	3d	5d	7d	0d	3d	5d	7d
0 mM	6.02	6.02	6.04	6.05	8.01	9.05	9.02	9.00	7.03	8.50	9.00	9.04
2 mM	6.02	6.00	6.00	6.00	8.01	9.00	9.02	9.06	7.03	7.30	7.38	7.46
5 mM	6.02	6.03	6.09	6.08	8.01	9.00	9.00	8.98	7.03	7.02	6.50	6.34
10 mM	6.02	6.00	6.02	6.00	8.01	8.78	7.63	7.11	7.03	6.80	6.23	6.00
15 mM	6.02	6.00	6.05	6.00	8.01	8.26	7.41	6.56	7.03	6.05	5.98	5.90

**Table 2:** O_2_ content (mg/L) in *P. sativum* experimental systems exposed to a series of Zn concentrations. Samples were analyzed at 0, 3, 5, and 7 days of co-cultivation.

O_2_	*P. sativum*/ Knop				*P. sativum*/ *Coelastrella* sp. BGV				*P. sativum*/ *Arthronema africanum*			
ZnSO_4_	0d	3d	5d	7d	0d	3d	5d	7d	0d	3d	5d	7d
0 mM	6.47	6.45	6.38	6.30	6.43	7.02	7.78	8.70	6.58	6.67	6.70	7.50
2 mM	6.47	6.45	6.37	6.30	6.43	6.58	6.65	7.44	6.58	7.19	7.59	7.89
5 mM	6.47	6.30	6.25	6.20	6.43	6.45	6.48	6.50	6.58	6.85	7.06	7.31
10 mM	6.47	6.26	6.18	6.09	6.43	6.31	6.23	6.10	6.58	6.41	6.11	6.00
15 mM	6.47	6.43	6.25	6.02	6.43	6.34	6.22	6.00	6.58	6.11	5.37	5.22

**Table 3:** Accumulation of Zn (g/kg DW) in the biomass of *Coelastrella* sp. BGV on the 7th day. Other elements are shown for comparison.

ZnSO_4_	P %	Fe, mg/kg	Cu, mg/kg	Mn, mg/kg	Zn, g/kg	Mg, mg/kg	Ca, mg/kg
0 mM	1.64	3748	70.1	2.16	**1.74**	8241	950
2 mM	2.19	2527	49.4	3.57	**106.11**	4514	693
5 mM	2.26	2214	45.9	0.96	**127.60**	4505	519
10 mM	1.33	1611	36.3	<0.2	**139.58**	2204	365
15 mM	1.18	1238	30.7	<0.1	**143.14**	1425	138

**Table 4:** Accumulation of Zn (g/kg DW) in the biomass of *Arthronema africanum* on the 7th day. Other elements are shown for comparison.

ZnSO_4_	P %	Fe, mg/kg	Cu, mg/kg	Mn, mg/kg	Zn, g/kg	Mg, mg/kg	Ca, mg/kg
0 mM	2.59	4094	346	6459	**1.51**	6483	774
2 mM	6.56	2483	233	4308	**134.42**	4125	425
5 mM	7.87	2768	298	3372	**166.90**	1580	274
10 mM	7.79	2880	273	2733	**169.73**	955	210
15 mM	7.81	3707	262	2327	**189.47**	850	262

**Table 5:** Accumulation of Zn (g/kg DW) in aerial parts and roots of *P. sativum* at day 7.

	*P. sativum*/ Knop		*P. sativum*/ *Coelastrella* sp. BGV		*P. sativum*/ *Arthronema africanum*	
	Zn, g/kg in *P. sativum*					
ZnSO_4_	Aerial Parts	Roots	Aerial Parts	Roots	Aerial Parts	Roots
0 mM	0.14	3.77	0.19	0.74	0.13	1.73
2 mM	0.48	9.93	0.38	12.68	0.43	23.96
5 mM	5.05	13.74	4.16	24.61	4.06	35.62
10 mM	2.25	18.47	0.84	11.59	0.26	19.26
15 mM	8.14	24.21	5.72	37.83	10.95	49.08

## Data Availability

The data presented in this study are available in this article.
